# Drug-Eluting Stents in Multivessel Coronary Artery Disease: Cost Effectiveness and Clinical Outcomes

**DOI:** 10.1155/2012/679013

**Published:** 2012-12-17

**Authors:** Kanaiya Panchal, Snehal Patel, Parloop Bhatt

**Affiliations:** ^1^Department of Pharmacology, Nirma University, Ahmedabad, Gujarat 382 481, India; ^2^Department of Pharmacology, L. M. College of Pharmacy, Ahmedabad, Gujarat 380009, India

## Abstract

Multivessel coronary artery disease is more often treated either with coronary artery bypass surgery (CABG) or percutaneous coronary intervention (PCI) with stenting. The advent of drug-eluting stent (DES) has changed the revascularization strategy, and caused an increase in the use of DES in multivessel disease (MVD), with reduced rate of repeat revascularization compared to conventional bare metal stent. The comparative studies of DES-PCI over CABG have shown comparable safety; however, the rate of major adverse cerebrovascular and cardiac events and repeat revascularization was significantly higher with DES-PCI at long term. In diabetic patients with MVD, concern of repeat revascularization with DES-PCI is persistent. More recent, one-year economic outcomes have reported that the CABG is favored among patients with high angiographic complexity. The higher rate of repeat revascularization with DES-PCI in MVD would lead to increased economic burden on patient at long term besides bearing high cost of DES. In diabetic MVD patients, CABG is associated with having better clinical outcomes and being more cost-effective approach when compared to DES-PCI at long term.

## 1. Introduction 

Coronary Artery Disease (CAD) is a major public health and medical concern in both developed and developing countries [1]. CAD, characterized by reduced blood supply to the heart muscle, is the most common cause of death in Western countries. The current treatment modalities for the coronary artery revascularization are Balloon Angioplasty, Percutaneous Coronary Intervention (PCI) with coronary stenting, and Coronary Artery Bypass Grafting (CABG) [2]. The choice of treatment modality is based on a various clinical characteristics, including patient age, comorbidities, extent and severity of disease, and number of diseased vessel, besides importantly lesion characteristics [3]. The optimal revascularization strategy for patients with multivessel coronary vessel disease remains a subject of debate. 

Earlier treatment of multivessel disease (MVD) was limited to balloon angioplasty or CABG, which then was managed with coronary stenting using Bare Metal Stent (BMS) with favorable clinical outcomes [4, 5]. Despite the benefits, use of BMS was hindered by the problem of restenosis owing to neointimal hyperplasia requiring repeat revascularization. In an effort to overcome the higher rate of restenosis with BMS, drug-eluting stents (DES) were developed [6]. The safety and efficacy of DES over BMS has been extensively investigated in a number of clinical trials and observational studies with reduced rate of redo interventions [7]. Although the indication of DES in MVD remains off-label and nonapproved by regulatory authorities (Per the Company DES Instructions for Use), the clinical safety of DES-PCI in MVD is increasingly evident. However, the significant higher cost of DES-PCI has raised the concern about its cost effectiveness over CABG in MVD patients. In light of the above facts, this paper summarizes the outcomes of contributing important clinical and pharmaco-economic coronary intervention studies comparing use of DES-PCI with CABG in MVD patients.

## 2. Balloon Angioplasty/Coronary Stenting versus CABG

- A number of studies have established the benefits associated with BMS over balloon angioplasty, prompting its use worldwide and by 1999, 84.2% of all interventions involved use of BMS [8, 9]. As compared to balloon angioplasty, BMS-PCI reduced the rate of repeat revascularization in MVD from 54% to 28% as reported in several studies like BARI, ERACI II, and ARTS trials [10–12]. These studies established the safety and effectiveness of BMS-PCI in MVD and encouraged its use in interventional practice.
- Several studies (Table 1) comparing BMS-PCI with CABG in MVD have shown favorable clinical outcomes with CABG, with lower rate of repeat revascularization at long term [11–13].
- Long-term safety profile, expressed by death, stroke, and myocardial infarction, was similar to that of CABG at 5 years (16.7% versus 16.9%, *P* < 0.69), but repeat revascularization were reported significantly more frequent after BMS-PCI than CABG (29.0% versus 7.9%, *P* < 0.001) in a pooled analysis of 4 randomized trials (ARTS, ERACI-II, MASS-II, and SOS Trials) [14].
- Long-term mortality at 5 years was similar after CABG and PCI (8.4% versus 10.0%) from collaborative analysis of individual patients with MVD, from six balloon angioplasty and four BMS trials. However, in subsets of patients older than 65 year and diabetic patients, reduced rate of mortality was reported following CABG as compared to PCI [15].
- Many studies have compared the cost effectiveness of both treatments in patients with MVD [16]. The incremental cost of CABG remained substantial when revascularization procedures were excluded from the cost analysis. In addition, the initial cost saving of 4,212 € with BMS compared to CABG decreased to 2,779 € after one year, 1,798 € after three years, and costs were similar after five years [17–19].

Thus, in MVD patients BMS-PCI reduces rate of repeat revascularization as compared to balloon angioplasty but has higher repeat revascularization as compared to CABG accounting for increased follow-up cost with similar long-term safety profile.

## 3. Drug-Eluting Stent in Real-World Setting

Substantial change in the revascularization practice of MVD was noted after the introduction of DES [7]. Structurally, DES combines different stent platforms (Stainless Steel, Cobalt Chromium) with various drugs (antiproliferative) and/or polymers having different elution rates and actions. The superiority of DES over BMS has been extensively studied in the form of Sirolimus Eluting Stent (SES) and Paclitaxel Eluting Stent in a number of studies [7, 20–22]. The second-generation DES, Zotarolimus (ENDEAVOR, Medtronic), and Everolimus (XIENCE, Abbott Vascular) also have been thoroughly investigated thereafter and confirmed superiority over BMS [23, 24]. By 2005 this resulted in use of DES in 80% to 90% of all revascularization procedures within United States [25]. However, it also warned against the risk of late stent thrombosis associated with DES [26], leading the US regulatory authorities to address the off-label use of DES [27]. Several individual reports and meta-analysis then confirmed the safety of DES in off-label indication in real-world practice [28, 29]. Newer DES with biodegradable polymer or DES that is polymer-free and completely biodegradable has been developed reporting exciting results. However, the clinical and cost effectiveness of newer DES in MVD is not adequately reported [30–33]. 

## 4. Drug-Eluting Stent in Multivessel Disease: Clinical Outcomes

Although historically CABG has been the treatment of choice in MVD, DES-PCI has been increasingly used in MVD as an off-label indication. The clinical safety and efficacy of DES-PCI over CABG have been examined in several studies in MVD patients mentioned below.

- Early studies like ARTS II and ERACI III have reported that DES-PCI was comparable to CABG with lower rate of repeat revascularization when compared with BMS cohort; however, the rate of repeat revascularization was reported higher as compared to CABG cohort [34, 35].
- In a meta-analysis of 24,268 patients with multivessel CAD treated with CABG or DES-PCI, overall Major Adverse Cerebrovascular and Cardiac Events (MACCE) were higher after DES-PCI due to excess of redo-revascularization compared with CABG at mean follow-up time of 20 months [36].
- More recent, results from multivessel revascularization registry of 5 years outcome of DES-PCI reported significantly higher rates of revascularization in DES-PCI group as compared to CABG, with similar rates of mortality and of the composite safety outcomes. This was also observed for diabetic subgroup [37].
- Comparision between PCI using Paclitaxel coated eluting stent and CABG (SYNTAX trial) was the largest randomized trial in 1800 patients, which assessed the optimal revascularization strategy in patients with three-vessel or left main coronary artery disease and enrolled more than 70% of multivessel CAD patients with or without left main disease. In MVD subset, the trial concluded that rate of MACCE was significantly higher in DES-PCI compared with CABG (28.8% versus 18.8%, *P* < 0.001), with significant difference in rate of revascularization (19.4% versus 10.0%, *P* < 0.001) at 3 years. The study also reported that in patients with less complex multivessel disease (low SYNTAX score), DES-PCI is an acceptable revascularization procedure (MACCE; PCI 28.8% versus CABG 22.2%, *P* = 0.45) [38, 39]. Extended followup at 4 years also reported lower rate of MACCE with CABG (PCI 33.5% versus CABG 23.6%, *P* < 0.001) in the overall cohort [40].

## 5. Diabetic Patients with MVD

- Diabetic subgroup trials reported that mortality and safety composite were comparable between the treatments, whereas revascularization was significantly higher in the DES-PCI as compared to CABG [41, 42]. Similar trend towards higher rate of repeat revascularization (28.0% versus 12.9%, *P* = 0.001) and MACCE (37.0% versus 22.9%, *P* = 0.002) with comparable safety endpoints (16.3% versus 14.0%, *P* = 0.53) was observed with DES-PCI in diabetic subgroup analysis of SYNTAX trial at 3 years [39].
- In 5-year followup of ARTS trial, results reported that CABG has comparable safety and superior efficacy compared to BMS and SES in diabetic patients with MVD, with significantly lower rate of repeat revascularization (SES 33.2% versus CABG 10.7%, *P* < 0.001) [43].
- Meta-analysis of studies comparing CABG with DES-PCI in diabetic patients with MVD have concluded that DES-PCI is safe, which may represent a viable alternative to CABG for selected diabetic MVD patients [44].
- FREEDOM trial was designed to determine the optimal revascularization strategy most recent to contemporary practice for the diabetic MVD patients and have addressed the clinical and cost effectiveness of DES-PCI over CABG at long term [45]. The results revealed the superiority of CABG over DES-PCI with reduced rates of death and myocardial infarction and higher rate of stroke in diabetic MVD patients at 5 year [46].

## 6. DES-PCI over CABG in Multivessel Disease: Cost Effectiveness

There has been continuous debate about which revascularization strategy amongst CABG and DES-PCI is more promising, as measured by overall cost effectiveness. The cost of a DES ($1,800 to $2,100) is at least three times higher than the price of a conventional BMS ($600 to $700) and most authors reported implantation of 3-4 DES per patient treated for MVD, leading to a significant increase in the initial total procedural cost [34, 38]. Numerous studies have established the cost effectiveness of DES over BMS in real practice [47–51], besides the favorable clinical outcomes with DES. These studies revealed significant reduction in the need for repeat revascularization procedures, leading to a decrease in the follow-up costs, in spite of higher cost of index procedure with DES as compared to BMS. 

Economic benefits of DES-PCI over CABG have been examined in very few studies that have enrolled MVD patients.

- A prospectively designed health economic evaluation embedded within the SYNTAX trial revealed that PCI using DES is an economically attractive strategy over the first year for patients with low and moderate angiographic complexity, while CABG is favored among patients with high angiographic complexity. The detailed cost analysis demonstrated that the procedural cost in PCI using DES were significantly higher ($14,509), owing to number of DES used (average 4.5 Stent), guide wires, balloon catheter, and medication cost, while overall initial hospitalization cost was $5,693/patient higher with CABG. At 1 year, follow-up cost was $2,282/patient higher with DES-PCI, mainly due to more frequent revascularization procedures. Finally, the total 1 year costs remained $3,590/patient higher with CABG, while quality-adjusted life expectancy was slightly higher with PCI. In a subset of patients with triple vessel disease, cost analysis revealed that the cost difference was 1,768/patient higher in CABG, with mean difference of repeat revascularization of 12.01 events/100 patients, and the incremental cost effectiveness ratio for CABG was reported $14,664 per repeat revascularization avoided. In addition, the complexity of MVD measured by SYNTAX score demonstrated that in patients with high angiographic complexity, total 1-year costs were similar for CABG and DES-PCI (difference of $466/patient), and the incremental cost-effectiveness ratio for CABG was $43,486 per quality-adjusted life-year gained [52].
- Besides, clinical outcomes and cost effectiveness, relief from angina and quality of life, may play a critical role in selecting a revascularization strategy. A sub-study of SYNTAX trial demonstrated that among patients with three-vessel or left main CAD, there was greater relief from angina after CABG than after DES-PCI at 6 and 12 months, although the extent of the benefit was small. The proportion of patients who were free from angina was similar in the two groups at 1 month and 6 months and was higher in CABG group than in the PCI group at 12 months (76.3% versus 71.6%, *P* = 0.05) [53].
- At 4-year followup it has been reported that, CABG is a more cost-effective strategy than DES-PCI in terms of preventing repeat revascularization, myocardial infarction and death, hence saving costs [54]. A cost effectiveness study of MVD in real-world setting reported a moderately higher rate of repeat revascularization and composite MACCE in DES-PCI compared to CABG, with significantly less costs over 5 years [55].
- According to very recent data, CABG was associated with higher initial cost (difference of $8,622) than PCI which subsequently reduced to $3,600 at 5 years due to higher follow-up cost with PCI in diabetic MVD patients [56].

## 7. Conclusion

According to most studies, the incidence of death/stroke/myocardial infarction in patients with MVD at long term was similar in CABG and DES-PCI. At long term, rates of repeat revascularization and MACCE are higher with DES-PCI than CABG. In diabetic MVD patients, similar outcomes were observed. However, the critical determining factor in MVD patients is the angiographic complexity. Based on current evidences, DES-PCI is an economically dominant strategy for patients with low and intermediate complexities while CABG is favored among patients with high angiographic complexities over first year in consideration of the clinical and cost effective outcomes. 

## Figures and Tables

**Table 1:** Death and repeat revascularization at five years with CABG and stenting.

Study Name	CABG versus Stenting	
	Rate of repeat revascularization	Death
ERACI-II	7.2% versus 28.4%, *P* = 0.0002	11.5% versus 7.1%, *P* = 0.182
ARTS	8.8% versus 30.3%, *P* < 0.001	7.6% versus 8.0%; *P* = 0.83
SOS	6.0% versus 20.7%, *P* < 0.001*	4.3% versus 8.1%; *P* = 0.016

*Clinical outcomes at median followup of 02 years.

## Abbreviations

ARTS: Arterial revascularization therapies study
ERACI II: Argentine randomized study: coronary angioplasty with stenting versus coronary bypass surgery in multivessel disease
BARI: Bypass angioplasty revascularization investigation
CARDia: Coronary artery revascularization in diabetes
FREEDOM: Future revascularization evaluation in patients with diabetes mellitus: optimal management of multivessel disease
MASS-II: The medicine, angioplasty, or surgery study
SOS: The stent or surgery trial
SYNTAX: Synergy between PCI with TAXUS and cardiac surgery.
